# Detection of copy number variations in rice using array-based comparative genomic hybridization

**DOI:** 10.1186/1471-2164-12-372

**Published:** 2011-07-20

**Authors:** Ping Yu, Caihong Wang, Qun Xu, Yue Feng, Xiaoping Yuan, Hanyong Yu, Yiping Wang, Shengxiang Tang, Xinghua Wei

**Affiliations:** 1 State Key Laboratory of Rice Biology, China National Rice Research Institute, Hangzhou, China

## Abstract

**Background:**

Copy number variations (CNVs) can create new genes, change gene dosage, reshape gene structures, and modify elements regulating gene expression. As with all types of genetic variation, CNVs may influence phenotypic variation and gene expression. CNVs are thus considered major sources of genetic variation. Little is known, however, about their contribution to genetic variation in rice.

**Results:**

To detect CNVs, we used a set of NimbleGen whole-genome comparative genomic hybridization arrays containing 718,256 oligonucleotide probes with a median probe spacing of 500 bp. We compiled a high-resolution map of CNVs in the rice genome, showing 641 CNVs between the genomes of the rice cultivars 'Nipponbare' (from *O. sativa *ssp. *japonica*) and 'Guang-lu-ai 4' (from *O. sativa *ssp. *indica*). The CNVs identified vary in size from 1.1 kb to 180.7 kb, and encompass approximately 7.6 Mb of the rice genome. The largest regions showing copy gain and loss are of 37.4 kb on chromosome 4, and 180.7 kb on chromosome 8. In addition, 85 DNA segments were identified, including some genic sequences. Contracted genes greatly outnumbered duplicated ones. Many of the contracted genes corresponded to either the same genes or genes involved in the same biological processes; this was also the case for genes involved in disease and defense.

**Conclusion:**

We detected CNVs in rice by array-based comparative genomic hybridization. These CNVs contain known genes. Further discussion of CNVs is important, as they are linked to variation among rice varieties, and are likely to contribute to subspecific characteristics.

## Background

Copy number variations (CNVs), or copy number polymorphisms (CNPs), are forms of structural variation (SV) that are alterations in DNA resulting in the cell having an abnormal number of copies of one or more segments of DNA. A CNV is a DNA segment ranging from 1 kb to 3 Mb that has been deleted, inserted, or duplicated, on certain chromosomes [1,2]. In particular, segmental duplications (SDs) were demonstrated to be one of the major catalysts and hotspots for CNV formation [3-5]. A CNV was described as early as 1936, with the duplication of the *Bar *gene in *Drosophila melanogaster *[6]. Recently, many studies have discovered CNVs in humans [7-9], chimpanzee [10], dog [11], cattle [12], rat [13], mice [14], *Drosophila *[15], yeast [16], *E. coli *[17], and maize [18,19]. CNVs can be detected using cytogenetic techniques such as fluorescent in situ hybridization, array-based comparative genomic hybridization, and SNP genotyping arrays. Recent advances in DNA sequencing technologies have further enabled the identification of CNVs by next-generation sequencing [20-22].

CNVs can create new genes, change gene dosage, reshape gene structures, and modify elements regulating gene expression [23,24]. Thus, CNVs are considered likely major sources of genetic variation, and may influence phenotypic variation and gene expression. Some human CNVs have been linked with susceptibility or resistance to disease. A higher *CCL3L1 *copy number, for example, can reduce risk of HIV/AIDS infection [25], and a lower *FCGR3 *copy number appears to contribute to increased susceptibility to glomerulonephritis [26]. CNVs also have an impact on fitness and gene expression. CNVs detected among 15 female isolines of *Drosophila *have been subjected to purifying selection [15]. In addition, a dramatic fruit size change due to a CNV with an insertion of 6-8 kb that affected gene regulation, was described during tomato breeding [27]. It was recently demonstrated that most CNVs in humans are in linkage disequilibrium (LD) with single nucleotide polymorphisms (SNPs); and that LD decay of the two happens at similar rates [8]. CNVs were confirmed to capture about 18% of the variation in gene expression, with little overlap with the variation captured by SNPs [28]. Thus, CNVs can be developed as a type of molecular marker for molecular identification.

Rice (*Oryza sativa *L.), comprises two subspecies, *indica *and *japonica*. It is one of the most important food crops in the world, and a model plant for genomic studies of monocots. Rice genomes exhibit relatively high levels of SNPs and indels [29]. Sequence comparisons between the Nipponbare (*japonica*) and 9311 (*indica*) genomes have shown high levels of polymorphisms ranging from one SNP/300 bp to one indel/kp [30,31]. These can potentially be exploited as molecular markers between these divergent subspecies. However, there are few studies of structural variation within the rice genome. Recent study of many subclones within chromosome 4 of the BAC libraries of Nipponbare and Guang-lu-ai 4 (*indica*), has documented that many genes vary in copy number [32]. With the completion of rice genome sequencing projects and advances in microarray technologies, comprehensive oligonucleotide microarrays are now being used to discover genetic polymorphisms. Array-based comparative genomic hybridization (aCGH) has the advantages of high resolution and high-throughput genome-wide screening of genomic imbalances, and has been used in rice to detect single-feature polymorphisms [33], and structural variations created by mutagenesis [34].

We used high-density oligonucleotide aCGH (containing 718,256 oligonucleotide probes) to investigate the number of CNVs between Nipponbare and Guang-lu-ai 4 genomes. We found high levels of CNVs, some representing large inserted/deleted regions. In addition, several DNA segments, often including genic sequences, were identified as present in the Nipponbare genome but absent from the Guang-lu-ai 4 genome. Ours is the first comprehensive map of CNVs in the rice genome; providing an important resource for understanding the nature of variation among different rice varieties.

## Results

### CNV detection using aCGH

**Figure 1:**
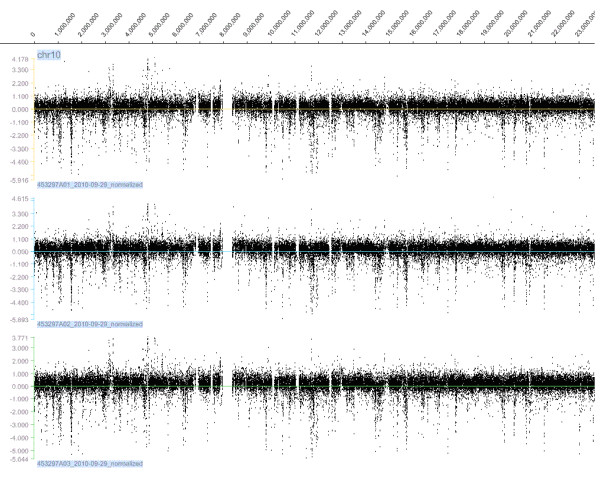
**An example of aCGH from our three replications**. The Y axis represents log_2 _ratios; the × axis represents genomic positions along chromosome 10.

**Figure 2:**
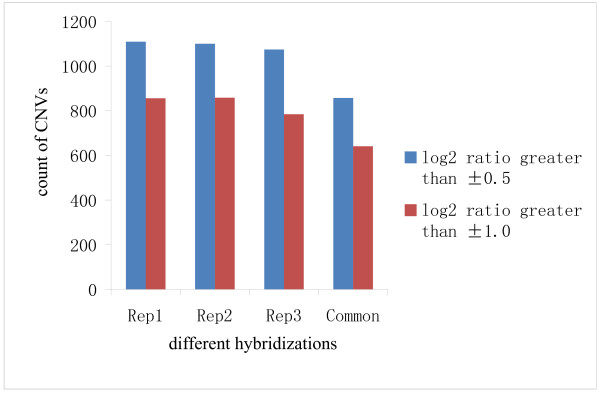
**Number of CNVs detected by aCGH in our three replications**.

To investigate the reproducibility of CNV detection using aCGH, we performed aCGH on three independent samples of Nipponbare and Guang-lu-ai 4 (Figure 1). In comparing hybridization results we decided that most detected CNVs may be accurate, even though some were not present in all replications. Using less stringent criteria, in which the log_2 _of the signal ratio between the two genomes was ± 0.5, we detected a total of 1,109, 1,100 and 1,074 CNVs respectively in three replications of Nipponbare and Guang-lu-ai 4; of which 857 (~78.3%) were detected in all three replications. However, using stringent criteria in which the log_2 _(Guang-lu-ai 4/Nipponbare) was ± 1.0, three comparisons of two samples revealed 856, 858 and 784 CNVs respectively; of which 641 (~77.0%) were detected in all three replications (Figure 2). Encouraged by this result, we surveyed hybridization signals which had high confidence levels and identified 641 CNVs.

**Figure 3:**
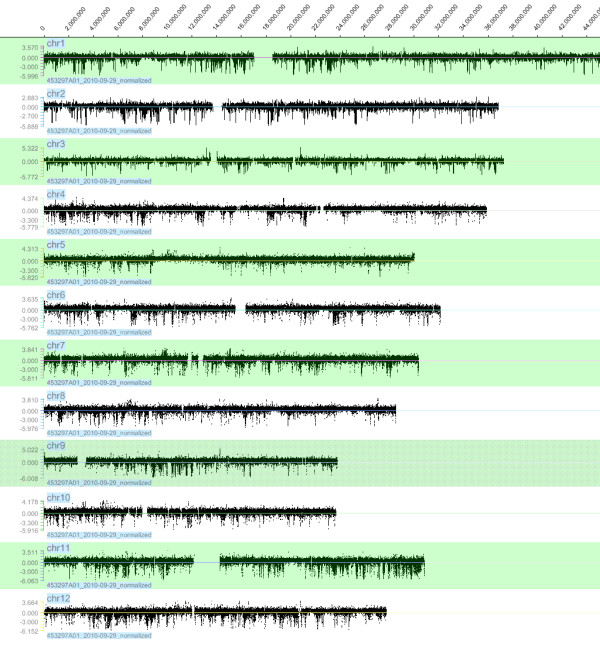
**Distribution of log_2 _(Guang-lu-ai 4/Nipponbare) signals throughout 12 chromosomes shown by aCGH analysis**. The Y axis represents the log_2 _of the signal ratio between Guang-lu-ai 4 and Nipponbare genomes; the × axis represents genomic positions along chromosomes.

**Figure 4:**
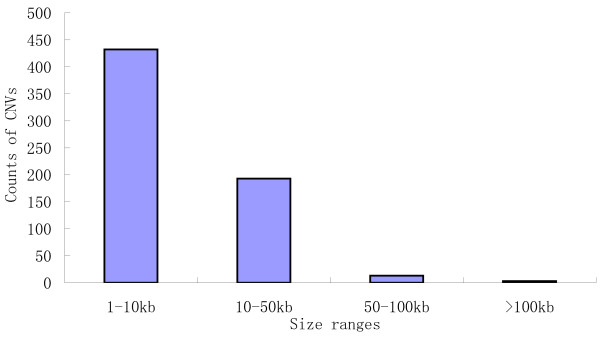
**Size range distributions of CNVs**.

These 641 CNVs comprised ~1.8% (~7.6 Mb) of the rice genome, similar to the proportion of CNVs in a population of *Drosophila melanogaster *(~2.0%) [15], and were distributed along all 12 rice chromosomes (Figure 3). We found no significant correlation between the frequencies of CNV occurrence and chromosome length (Additional file 1, Figure S1). The highest frequency (94) was found on chromosome 11, and the lowest frequency (30) on chromosome 5. This is consistent with a previous study of heterogeneous distribution of CNVs [35]. CNV sizes ranged from 1.1 kb to 180.7 kb, averaging 11.8 kb. Most CNVs (67.4%) were found to be small variants (< 10 kb), while some (2.5%) were larger variants (>50 kb) (Figure 4). The largest regions showing copy gain and loss were 37.4 kb on chromosome 4 and 180.7 kb on chromosome 8 (Additional file 2, Table S1). Analysis of the aCGH data also revealed a bias towards stronger hybridization signals from the Nipponbare genomic DNA than from the Guang-lu-ai 4 genomic DNA. This was found in CNVs determined by stringent criteria as well as those determined by less stringent criteria. This reflects the fact that the probes were designed from Nipponbare sequences.

### PCR analysis

**Figure 5:**
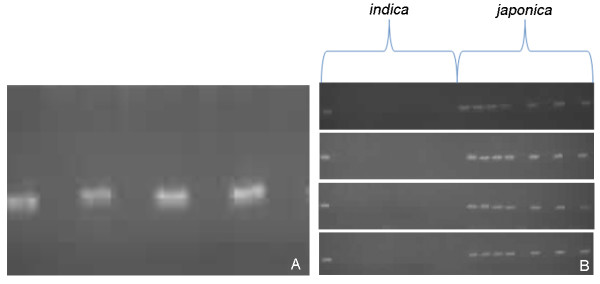
**PCR validation of a CNV identified by aCGH**. A) PCR amplifications for probes are shown in Nipponbare and Guang-lu-ai 4; B) PCR amplifications are shown 10 *indica *and 10 *japonica*.

We used 134 PCRs to further analyze 85 putative CNVs detected by aCGH. All PCRs confirmed the existence of insertion/deletion polymorphisms in these regions (Additional file 3, Table S2). More than 90% showed presence/absence variations between Nipponbare and Guang-lu-ai 4. All the validated CNV regions were defined by a few probes. In a CNV located on chromosome 12, for example, four amplicons spanning those probes of the putative deletions did not amplify from Guang-lu-ai 4 (Table 1), indicating that the DNA segment was absent from Guang-lu-ai 4 (Figure 5A). In addition, we also identified the allelic versions in 20 varieties of the two subspecies, and obtained similar results; more amplification products were present in *japonica *than in *indica *(Figure 5B). *Indica *and *japonica *are derived from independent domestication events of an ancestral rice that had already differentiated into two gene pools [36-38]. It seems unlikely that our observed pattern could be generated randomly, but our low number of samples prevents us from confirming strong evidence of subspecific variation in our CNV analysis.

**Table 1:** Primers used in PCR validation of a CNV located on chromosome 12 in Nipponbare, Guang-lu-ai 4, and some other varieties of *indica *and *japonica*.

Primer	Forward	Reverse
PP16-13477617	TGCGCTTCTTTGGCCTTCCGAT	TGAGCAAGCTGCGTACAAGGTT
PP16-13478047	GCATTGGGCTAAAAAGCAAGGCGC	TGGAGGCCCTCAAGCATATCCCA
PP16-13478457	TTGGACCTGCTGTGAGCCCGAT	ACCGCCTTTGGTCTCCCTCGTAC
PP16-13478857	GCTGCAAAGCGGACCCTAGCT	AGCTAATGATGGCTCACGAGAAGC

### Annotation of CNVs

Different hybridization signal intensity of a gene across aCGH would indicate a gain or loss of a gene copy number during rice evolution. Using a stringent selection criterion (a Guang-lu-ai 4 to Nipponbare signal ratio of 1 : 2), we identified 500 protein-coding genes that were contracted in Guang-lu-ai 4, and only 19 genes that were duplicated (signal ratio > 2.0) (Additional file 4, Table S3). The dominance of gene contraction over duplication was obvious when the aCGH selection ratio was relaxed (data not shown). Contracted genes thus greatly outnumbered duplicated ones. The majority of contracted genes are hypothetical proteins, indicating duplication of preexisting genes to augment gene function. Among the 19 duplicated genes, three encode different enzymes: transposase, reverse transcriptase and terpenoid cyclase. One gene is involved in gibberellin synthesis, i.e. ent-kaurene synthase like-2. *Xa1 *is a known bacterial blight resistance gene. Duplication also occurred in genes relating to metabolism, such as the GTP-binding signal recognition particle SRP54, and the 2-oxoglutarate dehydrogenase E2 subunit. As well, two genes were involved in transcription, the RNA polymerase III RPC4 family protein and the C2H2-type zinc finger domain-containing protein. Many of the contracted genes corresponded to genes that were either the same genes or genes involved in the same biological processes. This was similar for genes involved in disease and defense, such as most of them encode proteins with conserved nucleotide-binding sites (NBS) and leucine-rich repeats (LRRs). In addition, Cytochrome P450 and concanavalin A-like lectin/glucanase play crucial roles in defending plants from disease.

## Discussion

Using aCGH, we have generated the first map of CNVs in the rice genome. After very stringent filtering, 641 CNV events were identified between the two rice subspecies cultivars Nipponbare and Guang-lu-ai 4. This is likely to represent a very conservative estimate of the true number of CNV events in the rice genome. Focusing only on the unique sequences in our microarray will have potentially led to an underestimation of the number of CNV events. This is due to the selective omission or reduction of probe density in some CNVs enriched regions that contain segmental duplications and diverse repetitive sequences. In addition, our stringent CNV calling criteria restrained the detection of putative true CNVs. Differing probe densities, algorithms and statistical criteria used in the literature, complicate comparisons of rates of CNVs among different organisms [2,9-11,13,39]. Our data suggest that smaller CNVs (< 10 kb) are much more frequent than larger ones; this is supported by other studies [8,19]. However, using next-generation sequencing techniques would offer advantages over aCGH as DNA variations and recombination breakpoints would be directly detected [21,40-44].

CNV number differs between species. In mammals, the mean number of CNVs per individual has been found to range from 14 in macaques [45] to 70 in humans [9]. In maize, around 400 CNVs have been detected between two cultivars (Mo 17 and B 73) [18,19]. We observed many more CNVs between *indica *and *japonica*, the main reason for this was that we used subspecific samples. *Indica *and *japonica *diverged from their *O. rufipogon *ancestor between 200,000 and 400,000 years ago [37,46,47], and have richly diversified during the processes of domestication and selection. Both phenotypic and molecular studies have confirmed a relatively high level of differentiation between these two subspecies [48], suggesting great variation. This is also indicated by the lower numbers of deleted gene regions (ranging from 2 to 359) between 14 mutants and their wild type IR 64 of *indica *[34]. More recently, tiling oligonucleotide microarrays with 42 million probes, showed that an average of 1,098 CNVs comprising 0.78% of the human genome were validated between two individuals [49]. This was also found in a previous study [35], indicating that increased density and improved probe design will help us to better understand the roles of CNVs in organisms.

Although the presence and phenotypic effects of CNVs in plants have been little investigated on the genomic level, the nature of CNVs detected in maize suggests that they may have considerable impact on plant phenotypes, including disease responses and heterosis. We detected at least 519 genes in our high confidence CNV regions (Additional file 3, Table S2). However, it is likely that more genes are affected. We found that genes in many CNVs were involved in resistance, and that most of these encode proteins with conserved nucleotide-binding sites (NBS) and leucine-rich repeats (LRRs). NBS-LRR genes in plants tend to cluster at the same loci within genomes [50,51]. Similarly, both resistance genes and quantitative trait loci (QTL) are clustered in the rice genome [52,53]. In addition to its functional and agronomic importance, the NBS-LRR gene family has a structural role within the genome [54].

Previous research showed strong evidence that natural selection may shape CNVs, both in their patterns of polymorphism and their distribution within the genome [9,15]. Long-term purifying selection has changed quantitative traits, and it is possible that genomic variation in rice supplies source material for the generation of novel alleles. This implies that characterization of rice CNVs is far from perfect, and provides a comprehensive view of the polymorphic phase of CNVs.

## Conclusion

We have demonstrated that CNVs are able to be detected in rice using array-based comparative genome hybridization. These are likely to be linked with subspecific characteristics and to provide an important resource for understanding variation among different rice varieties.

## Methods

### Source of DNA samples

The rice varieties for our aCGH survey, Nipponbare (*japonica*) and Guang-lu-ai 4 (*indica*), were provided by the China National Rice Research Institute, Hangzhou, Zhejiang Province. The 10 *indica *varieties for CNV validation were: Minbeiwanxian, Dianbaidashanwang, Sankecun, Aizizhan, Haohuangla, Chiliyubai, Nanjing 11, Zhechang 9, Liantangao and Zhuguang 23. The 10 *japonica *varieties were: Kendao 8, Guihuahuang, Xiushui 48, Baimaodao, Xingguo, Mingshuixiangdao, Maendalaqili, Weiguo, Zhongdan 2 and Shuiyuansanbaili.

Genomic DNA was extracted and purified from fresh young leaves using a Promega kit (Wizard^® ^Genomic DNA Purification Kit). Total DNA was quantified using a spectrophotometer and electrophoresed on an agarose gel for integrity checking. Following the NimbleGen quality control requirements, the genomic DNA was undegraded and had 1.8 ≤ A260/A280 ≤ 2.0 and 1.9 ≤ A260/A230 ≤ 2.0.

### Array CGH

Custom NimbleGen 3 × 720 K microarrays http://www.nimblegen.com contain 718,256 oligonucleotide probes designed and fabricated on a single slide; resulting in a median probe spacing of 500 bp. These types of arrays utilize synthetic probes 45 to 75-mer in length with similar melting temperatures, and do not require sample amplification or reduced representation. Probes were designed from the NCBI rice genome build of October 2006. Roche NimbleGen's CGH probe design criteria was utilized. Uniqueness information was generated using the SSAHA program http://www.sanger.ac.uk/Software/analysis/SSAHA/. Standard genomic DNA labeling (Cy3 for samples and Cy5 for references), hybridizations, array scanning, data normalization, and segmentation were performed at CapitalBio Corporation as described previously [39,55]. High confidence calls were made according to the criteria used by Graubert et al. (2007). NimbleGen has an information package that describes the technology and provides measures of reproducibility, accuracy, sensitivity, and specificity. In brief, we used the normalized qspline method from the Bioconductor package in R. CNVs were identified by the circular binary segmentation algorithm [56]. Candidate CNVs were identified by finding more than 5 probe segments with log_2 _ratios greater than ± 1.0. We conducted further analysis and visualization using SignalMap software (NimbleGen). Raw aCGH data for this study have been deposited to GenBank GEO database under accession GSE30542http://www.ncbi.nlm.nih.gov/geo/query/acc.cgi?acc=GSE30542.

### Polymerase chain reaction (PCR)

For validation, sequences flanking the first and last probe set location of CNV regions were used to design primers. In addition, to reduce the possibility of interference from overlaps between probes and primer sequences, we designed two independent pairs of primers to confirm partial validated CNVs. PCR methods followed those recommended by the *TaKaRa LA Taq *manufacturer, optimizing conditions for each use. Products were run on a 1.5% agarose gel, stained with ethidium bromide, and visualized on a UV transilluminator.

## Abbreviations

CNV: Copy number variation; SV: Structural variation; SD: Segmental duplication; SNP: Single nucleotide polymorphism; LD: Linkage disequilibrium; aCGH: Array-based comparative genomic hybridization; NBS: Nucleotide-binding sites; LRR: Leucine-rich repeats; QTL: Quantitative trait loci.

## Authors' contributions

PY and XW conceived and designed the experiments. PY, CW, QX, and YF performed DNA preparations. HY and YW carried out microarray processing, and PY performed PCR analysis and contributed to interpretation of the data. PY, XW, and ST drafted the manuscript. All authors read and approved the final manuscript.

## Supplementary Material

Additional file 1 **Excel file includes Figure S1**. Correlation between chromosome length and number of CNVs. Click here for file

Additional file 2 **Excel file includes Table S1**. CNV regions detected and the sizes ranges of CNVs. Click here for file

Additional file 3 **Excel file includes Table S2**. Primers used for PCR validation of CNV regions. Click here for file

Additional file 4 **Excel file includes Table S3**. Genes included within CNV regions. Click here for file
